# Anatomic and Diagnostic Challenges of C-Shaped Root Canal System

**DOI:** 10.5005/jp-journals-10005-1230

**Published:** 2014-04-26

**Authors:** Deepak Raisingani, Shailendra Gupta, Prachi Mital, Poorva Khullar

**Affiliations:** Professor, Department of Conservative Dentistry and Endodontics Mahatma Gandhi Dental College and Hospital, Jaipur Rajasthan, India; Professor and Head, Department of Conservative Dentistry and Endodontics Mahatma Gandhi Dental College and Hospital, Jaipur Rajasthan, India; Postgraduate Student (1st Year), Department of Conservative Dentistry and Endodontics Mahatma Gandhi Dental College and Hospital, Jaipur Rajasthan, India; Postgraduate Student (2nd Year), Department of Conservative Dentistry and Endodontics Mahatma Gandhi Dental College and Hospital, Jaipur Rajasthan, India

**Keywords:** Tooth anatomy, C-shaped canal, Mandibular 2nd molar, Root canal treatment

## Abstract

Successful root canal treatment depends on the thorough management of the canal anatomy. The use of periapical radiographs is essential to identify and monitor the canal's morphological variations. The C-shaped single canaled man-dibular 2nd molar probably requires a different regimen of treatment from the two rooted, three canaled version, as it is rare. Because of the importance of its true diagnosis and treatment, a comprehensive review of published information and investigations about it in addition to approaches for its treatment is necessary. In this article, a detailed review and three case reports with different C-shaped canal configurations have been described which were successfully negotiated, pre­pared and obturated.

**How to cite this article: **Raisingani D, Gupta S, Mital P, Khullar P. Anatomic and Diagnostic Challenges of C-Shaped Root Canal System. Int J Clin Pediatr Dent 2014;7(1):35-39.

## INTRODUCTION

The crux of successful endodontics revolves around know­ledge, respect and appreciation for root canal anatomy and careful, thoughtful, meticulously performed cleaning and shaping procedures. Knowledge of pulpal anatomy, both the usual and unusual configurations and possible vari­ations is critical for success in endodontics and lack of such knowledge may lead to treatment failure.^1^

One of the most important anatomic variations is the ‘C’ configuration of the canal system. The C-shaped canal, which was first documented in endodontic literature by Cooke and Cox in 1979, is so named for the cross-sectional morphology of the root and root canal. Instead of having several discrete orifices, the pulp chamber of the C-shaped canal is a single ribbon-shaped orifice with a 180° arc (or more), which, in mandibular molars, starts at the mesiolingual line angle and sweeps around the buccal to the end at the distal aspect of the pulp chamber. Below the orifice level, the root structure can harbor a wide range of anatomic variations. These can be classified into two basic groups: (1) those with a single, ribbon-like, C-shaped canal from orifice to apex and (2) those with three or more distinct canals below the C-shaped orifice. Fortunately, C-shaped canals with a single swath of canal are the exception rather than the rule.^2^

## ETIOLOGY

The shape and the number of roots are determined by Hertwig's epithelial sheath, which bends in a horizontal plane below the cementoenamel junction and fuses in the center leaving openings for roots.^3^ Failure of the Hertwig's epithelial root sheath to fuse on the lingual or buccal root surface is the main cause of C-shaped roots, which always contain a C-shaped canal. The C-shaped root may also be formed by coa­lescence because of deposition of the cementum with time.^4-6^

## CLASSIFICATION

### Melton's Classification^7^

**Fig. 1:**
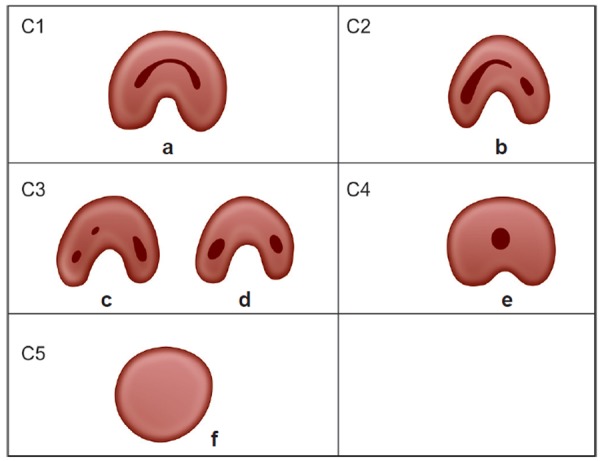
Classification of C-shaped root canal configuration according to melton

Melton et al in 1991 proposed the following classification of C-shaped canals based on their cross-sectional shape (Fig. 1):

**Fig. 2:**
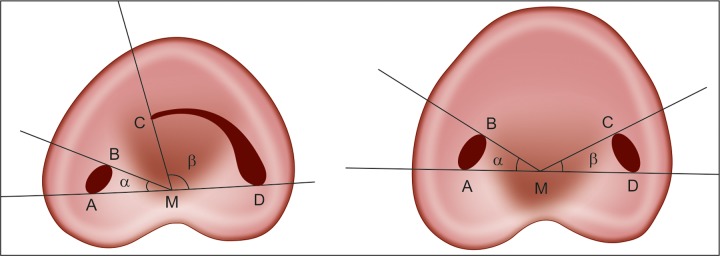
Classification of C-root canal configuration according to fan

**Figs 3A to C:**
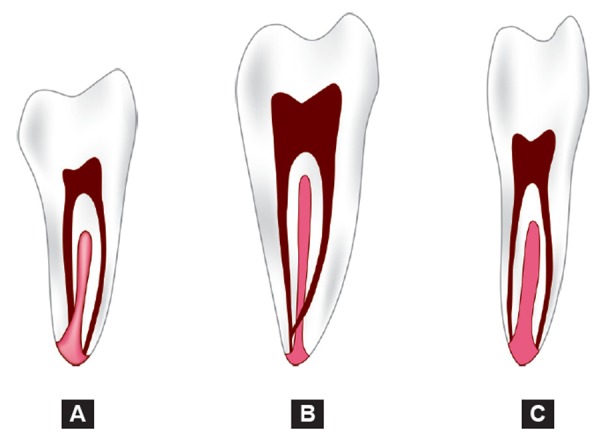
Fan's radiographic classification

**Fig. 4:**
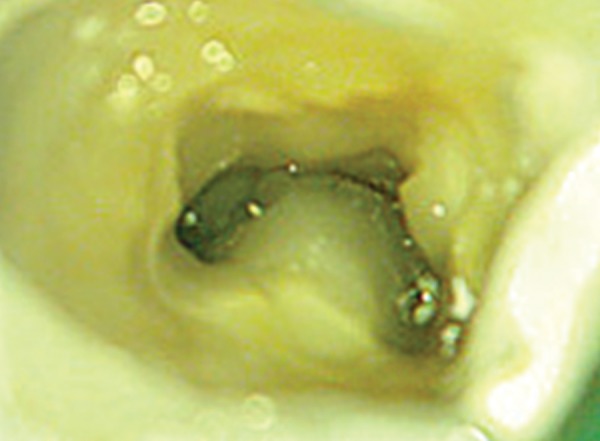
Clinical appearance of a C-shaped canal configuration in mandibular second molar

*Category I*: Continuous C-shaped canal running from the pulp chamber to the apex defines a C-shaped outline without any separation.

*Category II*: The semicolon-shaped orifice in which dentine separates a main C-shaped canal from one mesial distinct canal.

*Category III*: Refers to those with two or more discrete and separate canals:

- *Subdivision I*: C-shaped orifice in the coronal third that divides into two or more discrete and separate canals that join apically.
- *Subdivision II*: C-shaped orifice in the coronal third that divides into two or more discrete and separate canals in the midroot to the apex.
- *Subdivision III*: C-shaped orifice that divides into two or more discrete and separate canals in the coronal third to the apex.

Fan et al^8^ in 2004 modified Melton's method of classi-fication into the following categories (Fig. 2):

- *Category I (C1)*: The shape was an interrupted ‘C’ with no separation or division.
- *Category II (C2)*: The canal shape resembled a semicolon resulting from a discontinuation of the ‘C’ outline, but either angle or should be no less than 60°.
- *Category III (C3)*: Two or three separate canals and both angles, and were less than 60°.
- *Category IV (C4)*: Only one round or oval canal in that crosssection.
- *Category V (C5)*: No canal lumen could be observed (which is usually seen near the apex only).

### Fan's Classification (Radiographic Classification)^9^

Fan et al classified C-shaped roots according to their radio-graphic appearance into three types (Figs 3A to C):

1. *Type I*: Conical or square root with a vague, radiolucent longitudinal line separating the root into distal and mesial parts. There was a mesial and a distal canal that merged into one before exiting at the apical foramen (foramina).
2. *Type II*: Conical or square root with a vague, radiolucent longitudinal line separating the root into distal and mesial parts. There was a mesial and a distal canal, and the two canals appeared to continue on their own pathway to the apex.
3. *Type III*: Conical or square root with a vague, radio-lucent longitudinal line separating the root into distal and mesial parts. There was a mesial and a distal canal, one canal curved to and superimposed on this radiolucent line when running toward the apex, and the other canal appeared to continue on its own pathway to the apex.

**Figs 5A and B:**
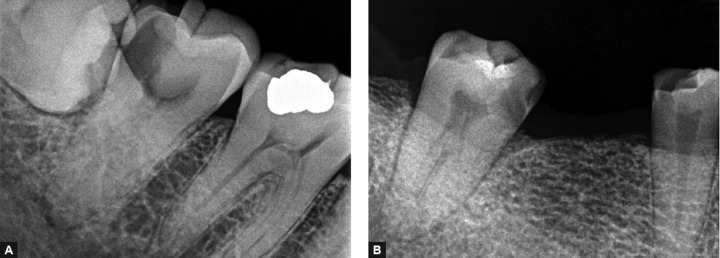
Radiographic appearance of a C-shaped canal configuration in mandibular second molar

## EPIDEMIOLOGY

The frequency of C-shaped canals varies greatly among different ethnicities. The prevalence of C-shaped canals in Asian populations has been reported to be as high as 30%. Gulabivala et al, in 2001, using a canal staining and tooth clearing technique, reported the incidence of 22.4% in Bur­mese patients.^10^ In 2002, using the injection of Indian ink, Gulabivala et al noted the prevalence of 10% in Thai popu­lation. At the same time, Al-Fauzan indicated the incidence of 10.6% in Saudi Arabian population.^11^

Endodontic textbooks state that the C-shaped canal is not uncommon and this is confirmed by studies in which frequencies ranging from 2.7% (16) to 8% have been reported. This configuration is a significant ethnic variation in the incidence of C-shaped molars. It is seldom found in white people and is much more common in Asians than in whites. This variation may occur in mandibular first molars, maxillary molars, mandibular first premolars, and even in maxillary lateral incisors, but it is most commonly found in mandibular second molars^2^ (Fig. 4).

When present on one side, a C-shaped canal may be found in the contralateral tooth in over 70% of individuals.^2^

## DIAGNOSIS AND MANAGEMENT

### Radiographic Diagnosis

The preoperative awareness of a C-shaped canal configuration before treatment can facilitate effective management. A preoperative radiograph and an additional radiograph from 20° mesial or distal projection may be the only noninvasive means clinically to provide clues about the canal morphology^9,12^ (Fig. 5).

Radiographs taken while negotiating the canals may reveal two characteristics for such canal configuration: instruments tending to converge at the apex and/or may exit at the furcation. The latter sometimes may resemble a perforation of the furcation.^2^

This radiographic appearance is more likely to occur in category I (continuous). The presence of instruments or filling materials in the furcation area in combination with the poorly distinguished foor of the pulp chamber can lead to radiographic recognition of ‘C’ configuration.^13^

Radiographic interpretation is overall more effective when based on flm combinations (‘preoperative and working length radiographs’ or ‘preoperative and final radiographs’ or ‘all three radiographs’) than on single radiographs. Among the latter, working length radiographs are more helpful than the preoperative and final ones, whereas preoperative radio­graphs are the least effective in diagnosing the C-shaped cases.^14^

**Fig. 6:**
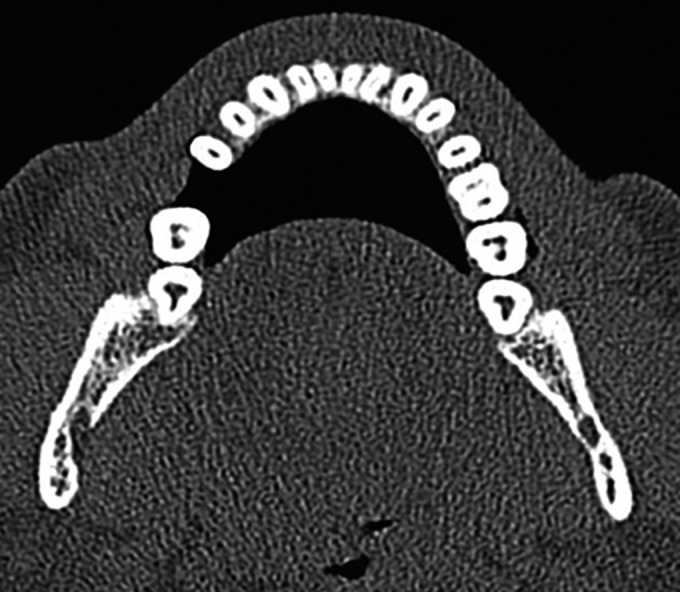
CBCT image of C-shaped canal system in both mandibular left and right molar teeth

Recent research used a spiral computed tomography (Fig. 6) scan to diagnose the canal anatomy, but the disso­lution of the image is not yet high enough to show irregular or fine canal structures, and the exposure to the relatively high dosage of X-ray radiation is also another concern. Although the radiographic contrast medium can enhance the radiopacity of target tissue images against other surrounding tissues, sometimes the enhancement is insufficient to make those thin, fine, or small structures discernible.^15^

## CLINICAL DIAGNOSIS

The pulp chamber in teeth with C-shaped canals may be large in the occlusoapical dimension with a low bifurcation. Alternatively, the canal can be calcified, disguising its C-shape. At the outset, several orifices may be probed that link up on further instrumentation (Fig. 5). In a true C-shaped canal, it is possible to pass an instrument from mesial to distal aspect without obstruction.^2^

## MANAGEMENT

### Canal System Identification and Preparation

The access cavity for teeth with a C-shaped root canal system varies considerably and depends on the pulp morphology of the specific tooth. Initial canal-system recognition occurs after achievement of routine endodontic access and removal of tissue from the pulp chamber.^13^

If a C-shaped root is present, two of Melton's three categories (category I and II) should be evident (in category III, two or three separate canals may appear initially as a typical three-canal orifice mandibular molar). In all categories, the mesiobuccal and distal canal spaces usually can be prepared normally. Also, Gates-Glidden burs should not be used to prepare the mesiobuccal and buccal isthmus areas.^7^

Because of the large volumetric capacity of the C-shaped canal system, housing transverse anastomoses and irregularities and continuous circumferential fling along the periphery of the C canal is irrigated with copious amounts of 5.25% NaOCl are often necessary to ensure maximum tissue removal and cessation of bleeding. HAFI (Hedstrom files) are especially effective for Efficient tissue removal. If hemorrhaging persists, then ultrasonic removal of tissue or placement of calcium hydroxide may be used between appointments to enhance tissue removal and control hemorrhage.^16^

Alternative canal cleaning techniques, such as those that use ultrasonics, would be more effective. An increased volume of irrigant and deeper penetration with small instruments using sonics or ultrasonics may allow for more cleansibility in fan-shaped areas of the C-shaped canal.^7^ Although ultrasonic preparation may effectively remove tissues from narrow C-shaped canal ramifications, aggressive instrumentation may cause perforation.^13^

## CANAL SYSTEM OBTURATION

Obturation of C-shaped canals may require technique modifications. The mesiolingual and distal canal spaces can be prepared and obturated as standard canals. However, sealing the buccal isthmus is difficult if lateral condensation is the only method used. Because this isthmus may not be prepared with a sufficient fare to permit deep placement of the spreader, application of thermoplasticized gutta-percha is more appropriate.^13,17,18^

## ENDODONTIC SURGERY

The clinician must be aware of the impact this anatomy has when surgical endodontics is indicated. The absence of furca contraindicates hemisection or root amputation. The intercanal communications or fins visualized on the serial sections reinforce the difficulty the clinician would encounter after apicoectomy with the retropreparation and eventual retrofilling. If endodontic surgical intervention is indicated for a molar with C-shaped root canal anatomy, strong consi­derations should be given to extraction, retrofilling, and intentional replantation.^19^

## RESTORATION AND PROGNOSIS

Technique modification may be required for restoration of C-shaped roots. If post placement for a crown core is desired, use of only the distal canal should be considered. Proper post-canal adaptation and stress distribution is more likely to result in the tubular distal canal. Placement of posts or antirotational pins in the mesiolingual and mesiobuccal areas of C-shaped root invites perforation. Also, post width should be minimized. It should be remembered that there is a higher risk of root perforation at the thinner lingual walls of C-shaped canals during shaping and post space preparation procedures. Both buccal and lingual canal walls are frequently narrower at mesial locations.^20^ During follow-up radiographic examination, the dentist should look for furcal breakdown because that region is the most difficult to obturate and is associated with the greatest risk of perforation. Restorations with failure in the furca have a poor prognosis. If the failure results from an apical etiology and apical surgery is not possible, viable options include extraction, extraoral retrofilling and replantation. Because C-shaped roots generally are conical, they are easy to extract without fracture. When sound principles of cleaning and shaping, obturation and restoration are followed, the long-term prognosis for the C-shaped root retention equals that of other molars, but cautious optimism would seem most appropriate when prognosticating the success of the root canal treatment of a C-shaped canal.^13^

## CONCLUSION

The early recognition of these configurations facilitates cleaning, shaping and obturation of the root-canal system. It should be noted that using a radiograph showing files set to the canal terminus to diagnose and to determine canal morphology may not give the results expected. In some instances, it may be difficult to distinguish between C-shaped canal or one with single or three canals joining apically. Thus, it is necessary to confirm the diagnosis by exploring the access cavity. Knowledge and recognition of canal configuration facilitates more effective canal identification and unnecessary removal of healthy tooth structure in an attempt to search for missing canals. All the cases were treated in multiple sittings to rule out the possibility of a missed canal.

Further long-term clinical studies are needed to sub­stantiate the diagnosis of this variant in mandibular second molars using various diagnostic methods for a better under­standing of this variant to facilitate cleaning, shaping and obturation of the root canal system.
